# Linking doctor-patient relationship to medical residents’ work engagement: The influences of role overload and conflict avoidance

**DOI:** 10.1186/s12875-021-01541-6

**Published:** 2021-09-24

**Authors:** Guangwei Deng, Wenjun Cai, Monica Yang, Jonathan Lio, Chenpeng Feng, Xiaopeng Ma, Liang Liang

**Affiliations:** 1 School of Management, Hefei University of Technology, Hefei, Anhui Province 230009 P.R. China; 2 School of Management, University of Science and Technology of China, Hefei, P.R. China; 3 Robert B. Willumstad School of Business, Adelphi University, Garden City, USA; 4 Department of Medicine, University of Chicago, Chicago, USA; 5 Department of General Surgery, the First Affiliated Hospital of University of Science and Technology of China, Hefei, P.R. China

**Keywords:** Doctor-patient relationship, Work engagement, Role overload, Conflict avoidance

## Abstract

**Background:**

Chinese residents’ practical work experiences are different from those described in Western studies. To explore potential mechanisms underlying the effects of doctor-patient relationships on medical residents’ work engagement, verifying a posited mediating effect of role overload, and moderating effect of conflict avoidance, in the Chinese context.

**Methods:**

Based on the conservation of resources theory, a composite model was constructed. This study’s data were collected from four different Chinese tertiary hospitals; 195 residents undergoing regularization training took this survey. Hierarchical moderated and mediated regression analyses were utilized.

**Results:**

Doctor-patient relationship were found to be positively related to residents’ work engagement (β=0.31, *p*≤0.001). Role overload partially mediated the effect of these relationships on work engagement, and the moderating role of conflict avoidance in the relationship between doctor-patient relationship and conflict avoidance was negative.

**Conclusion:**

Maintaining good doctor-patient relationship can prompt residents to increase their engagement in work in order to meet their patients’ needs. Furthermore, role overload has a particular influence in early career stages. Not only is it necessary for residents to gain a sense of recognition and support while they carry out their job responsibilities, especially while dealing with complex doctor-patient relationship, but it is also important to create work environments that can help residents shape their professional competency.

## Introduction

The past five to ten years have witnessed the emergence of a wealth of human resource management research focusing on work engagement, which has demonstrated that work engagement is positively related to enhancing performance and achieving goals [1, 2]. Kahn (1990) originally pioneered the concept of personal engagement with work as employees “bring in” their personal selves to their work role, engaging and expressing personal physically, cognitively and emotionally during role performances [3], and came to be recognized as an organizational element that counteracts job burnout and is closely related to positive organizational results [4]. More work engagement makes work state more effective: higher efficiency, vigor, dedication, focus and flow state, as well as lower turnover intention [5–8]. Thus, we hold that work engagement is a key factor ensuring the success of health organizations and the lasting competitiveness of medical professionals [1, 9]. This makes it a critical issue in medical education research. The challenges raised by high burnout rates during medical residency training in various countries highlight the importance of ensuring high work engagement, harmonious doctor-patient relationship, and reasonable role positioning and task allocation within a complex medical environment [10, 11]. There have been impressive advances in the empirical and theoretical research on work engagement; however, certain aspects remain to be understood, which could lead to a better understanding of its underlying mechanisms. For example, research on the mechanisms underlying work engagement in the health field is insufficient, particularly regarding effective work engagement among front-line medical staff [12]. From the perspective of residents’ experiences, work engagement-related research involving factors operating between individuals and work situations are still lacking, including doctor-patient conflicts, role overload, conflict avoidance, and so on.

In China, the large, rapidly aging population has strained medical resources; the full liberalization of the former two-child policy and growing awareness of increasingly affordable access to public health care have exacerbated this problem [13]. Given the special conditions in China, Chinese residents’ practical work experiences are different from those described in existing, large Western studies [14]. Chinese residents experience workload and thus they are facing time pressure and stress [15]. Furthermore, because the Chinese standardized training system is still in an exploratory process, role overload often occurs because of residents’ unclear role positioning and unreasonable division of labor (see “The mediating effect of role overload” section). Like most Asian cultures, China deeply reveres values such as harmony, collectivism, and social dedication, and Chinese people also support strict government control of public hospitals. In many cases, fearing negative effects on their future careers, residents deliberately avoid all kinds of potential doctor-patient conflicts, including those occurring due to opposition to hospital policies. Therefore, we introduced the variable of conflict avoidance (see “The moderating effect of conflict avoidance” section), which Chinese residents may find important to help them deal with doctor-patient relationship and tasks.

**Fig. 1:**
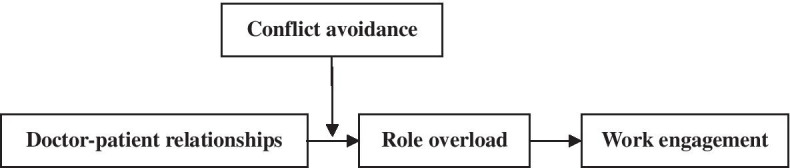
Holistic hypothetical model

To investigate these issues, we adopted the conservation of resources theory and proposed a theoretical model that tried to explore the potential mechanisms underlying the effect of doctor-patient relationship on residents’ work engagement from the perspective of residents and used an empirical research method; in addition, we attempted to verify the mediating effects of role overload and the moderating effects of conflict avoidance in the model (See Fig. 1).

## Theoretical background and hypotheses

### Doctor-patient relationship and work engagement

Work engagement can be broken down into emotional or psychological engagement, essentially the degree to which the worker wants to contribute, and actual engagement, or the actual energy and effort they do contribute and the results. Both are directly linked to residents’ performance [16, 17]. In addition to their work attitudes, we need to understand residents’ attitudes toward doctor-patient relationship [18]. In most countries, doctor-patient relationship is considered more important than any other social relationships except familial relationships [19]. Residents are the direct suppliers of medical services and reliable sources of health information. Thus, the status of the relationships with their patients influences the high-quality medical care provision that patients experience. Family members are also vital stakeholders because, in most cases, they are the patients’ chief supporters and often decision-makers [20, 21]. Thus, the relationships between doctors and patients’ families also play a key role in treatment and care experience.

There are several studies using doctor-patient conflict to represent the doctor-patient relationship, because conflict is an utmost important relational outcome, especially in the context of Chinese medical treatment [20, 22, 23]. The degree of conflict with patients (and their families; henceforth, however, “doctor-patient relationship”) can deeply influence residents’ future career lives. Training residents to pay greater attention to patients and avoid conflict with them during the early periods of their careers could be very beneficial for the patients, the residents themselves, and the medical system as a whole [18]. With experience, residents may be able to better perceive the degree of doctor-patient conflicts and the status of doctor-patient relationship; this could considerably influence whether the behavioral strategies they adopt will be patient-centered or doctor-centered. In patient-centered behavioral strategies, doctors prioritize a more humanistic spirit, develop greater work engagement, and proactively provide medical services that can best meet their patients’ needs; this results in higher patient satisfaction [24–26]. In contrast, doctor-centered approaches are paternalistic in nature; in this approach, doctors are more likely to emphasize their own interests and take defensive medical actions to protect themselves. During the diagnosis and treatment processes, they are more likely to have ideas that differ from those of their patients, especially with regard to certain medical behaviors, methods, attitudes, and consequences; thus, this approach produces a greater degree of doctor-patient conflict [18].

The conservation of resources theory holds that groups with fewer occupational resources [27], such as beginner residents, are extremely sensitive to occupational resource depletion. The gain spiral of resources principle [28, 29] holds that if residents maintain good relationships with patients, they will receive greater support and recognition, ideally yielding a conditional resource that benefits their work. At the same time, recognition received from patients and their families functions as a kind of professional stimulation that can promote residents’ sense of achievement and self-satisfaction, which can also be counted as (“personal characteristic”) resources. Residents possessing more resources are more likely to acquire new resources and acquire added value of resources. Investing in these resources, they may counteract resource loss, and as a result may become more dedicated and increase their work engagement. According to previous research [30, 31], good doctor-patient relationship can be considered as to be a job resource, which is able to activate a motivational process that contributes to the achievement of training goals, it can avoid costly psychological and physical costs, foster residents’ growth, increase career possibility, provide opportunities for residents to learn; and satisfy needs for clinical work autonomy and competence [30, 31]. Good doctor-patient relationship can also increase individual willingness to devote one’s efforts and abilities to the training task, and these perceptions and beliefs increase the degree to which residents are willing to invest their selves into their role performances [30, 31].

Thus, good doctor-patient relationship (that is, lower conflict rate between doctors and patients as well as their families) should positively predict residents’ work engagement.

#### Hypothesis 1 (H1)

Doctor-patient relationship positively influences the work engagement of residents.

### The mediating effect of role overload

Role overload describes a condition under which individuals lack the time or energy to meet their role expectations (whether those expectations originate in others or in oneself) [32]. In hospital settings, “role senders” or deciders of residents’ roles are typically supervisors or clinical teachers. Because they work at the frontlines of clinical practice, residents have high contact frequency with patients [33]; thus, their roles are also affected by the requirements of and feedback from patients and their families: that is, their role expectations of the residents. Thus, residents must also undertake tasks unrelated to their training [34]; for example, in some tertiary hospitals lacking adequate beds, patients may seek the assistance of residents they have good relationships with to arrange beds for them in advance.

Most research opinions in the past have indicated that, from an organizational management perspective, role overload is harmful, causing stress, exhaustion and degrading care [35, 36]. In contrast, well-controlled and -defined roles and adequate discretionary rites can alleviate these difficulties and even stimulate residents to work harder, raising their work engagement [37]. The biggest feature of workplace learning is “learning by doing” [38]; in medical environments, residents may experience role overload because they often aim to gain more professional training and experience accumulation outside the formal scope of their training [39], but at the same time, this may be the only way for them to gain certain kinds of experience such as experience with ethical issues and may be effective to help them build their professional reputation [40].

In addition, the mediation function of role overload may be especially large at the resident stage, the “establishment stage” of this career, which compared to ‘maintenance” and “disengagement” stages is marked by the need to root oneself, learn and become known (the “grounding” process) and hence by less concern among residents for role overload, as they are more focused on achieving success. Role overload may be perceived as less a burden than a challenge or necessary sacrifice, according to Jones et al. [41], residents who are in this period are usually eager to build their reputation in medical industry by achieving good performance, in most cases, role overload is considered as necessary sacrifice to gain achievements.

#### Hypothesis 2 (H2)

Role overload mediates the relationship between doctor-patient relationship and the work engagement of residents.

### The moderating effect of conflict avoidance

Conflict avoidance involves refusing to engage in any conflict and, furthermore, positively engaging in actions to solve conflict [42]. The concept of conflict avoidance is highly relevant in many Asian cultures, and especially in Chinese culture [43, 44]. The Chinese Confucian tradition holds harmony to be a core value and thus encourages people to tolerate interpersonal disagreements and transgressions [45]. Conversely, pursuing conflicts with others can cause individuals to lose face in such societies, and losing face, in turn, can bring shame to themselves and others [46]. Thus, conflict avoidance helps to protect a relationship between two parties of conflict who may have differing opinions; it lowers the possibility of aggression from either side and maintains the positive images of all involved parties [44, 47]. Thus, Chinese people tend to do their best to maintain harmonious working relationships, viewing this as a mature behavior with high moral value.

In Chinese traditional culture, doctors are expected to meet high role expectations; to be considered outstanding, doctors must have high moral standards. Most Chinese people have deeply embedded this mode of value recognition. Many young residents also perceive their careers to have high role requirements and expectations; they believe that having conflicts with patients and their families indicates a lack of professionalism and moral spirit [48]. It could also affect their professional reputation and future career development, so their awareness of conflict avoidance is very strong. However, some residents show a very weak awareness of conflict avoidance; reasons for this variation include individual- and cultural-level differences and lack of communication and interpersonal skills [48–50]. In situations where they face emotional challenges, some residents are unable to access or use effective emotional management strategies, and it is easy for them to experience conflicts with patients and patients’ families [51]. Thus, in this study, the degree of conflict avoidance among residents showed variability.

As stated previously, according to conservation of resources theory [27], conflict avoidance, as a measuring standard for cultural values, moderates the relationship between residents’ conflicts with patients and role overload in the Chinese context. Individuals vary in sensitiveness to resource losses: those who have fewer resources are more likely to view the resources they do have as precious and to do their best to avoid losing their resources; however, usually, they are unable to do so, and they may even end up losing more resources [28, 52]. For instance, some residents, who may not be skillful at avoiding conflicts, may attempt to control their emotions in order to improve doctor-patient relationship in the long term; however, these residents may consequently face more negative emotions from themselves as a result of their efforts at self-control. In this way, decreasing conflict may thus lead to resource loss and greater role overload.

#### Hypothesis 3 (H3)

The effect of doctor-patient relationship on role overload is negative moderated by conflict avoidance.

### Samples and procedures

This study’s data was collected as part of a larger questionnaire survey, which was administered to residents in five tertiary hospitals (as only such hospitals provide residency training in China) located in the southeast and central regions of China. Our study included residents from the following clinical departments: internal medicine, surgery, pediatrics, obstetrics, community medicine, intensive care unit (ICU), emergency, psychiatry, and so on. The survey instrument included a large number of variables related to residents’ attitudes and perceptions, which allowed us to test the above-developed hypotheses. First, we explained the background and goals of our research to residents’ managers or informal resident organizations in hospitals via in-person visits, phone, or social networking platforms (e.g., QQ, WeChat). After receiving these organizations’ approval, we started our field investigation. Participation in this research was voluntary, and anonymity was assured to all participants. The survey interviews happened during residents’ off hours, lasting for an average of 20 minutes, each questionnaire concludes 70 questions and it covered residents’ attitudes, perceptions and their background information. In addition to 31 questionnaires that were later mailed to the researchers, 141 were collected on site. Of these 195 questionnaires (65% response rate), 23 were discarded because of data missing. We examined the differences of resident gender, age, marriage, education, and doctor qualification between the valid participants and the discarded samples, and the results of t-test showed no significant difference.

The demographic profiles of the 172 valid participants are presented in terms of the following demographic variables: gender, age, education, and teachers rank. The average age of respondents was approximately 30 years, 54.1% were female and 45.9% male. Of the respondents, 33.7% were married and 71.5% had passed the medical qualification exam. Participants also reported that 47.6% of them had a bachelor’s degree and 51.7% had a master’s degree.

### Variable measurement

All the measurement items were evaluated at the individual level using a 5-point Likert-type scale ranging from 1 (strongly disagree) to 5 (strongly agree). The scales were translated from English to modern Mandarin, and retranslating the final version back into the original language. This procedure enabled a group of experts to ensure the accuracy of the meaning.

#### Work engagement of residents

Work engagement was measured with a nine-item scale adapted from Thomas [53]. An example item from the scale is “I am willing to really push myself to reach challenging work goals”.

#### Doctor-patient relationship

This variable was assessed with three items developed by Lee et al. [20]. We measured the degree of differences and disagreements between residents and patients on personal issues, patient care issues and the way work is done. A sample item included “For residents and patients or their families, how would you rate the degree to which there are disagreements over personal issues”.

#### Role overload

It was measured with the unidimensional scale of role overload [32]. It consists of six items. An example item is “I have to do things that I do not really have the time and energy for”.

#### Conflict avoidance

We adopted the scales of Park and Nawakitphaitoon [43], which is based on the research of Morris [54]. An example item is “I believe it is better to keep negative opinions to ourselves rather than create hard feelings”.

#### Control variables

Following Becker et al.’s [55] recommendation, we controlled five variables that are theoretically related or have been found to be empirically related to residents. For examples, gender was measured as 1=Female, 2=Male; education level indicates the degree level of residents, including 1=below college diploma, 2=college diploma, 3=bachelor, 4=master and above; marriage was operated as 1=Yes, 2=No.

## Results

### Reliability and validity

For all multi-item constructs, the reliability and validity of measurements were evaluated using confirmatory factor analysis (CFA). Table 1 shows the CFA results. The Cronbach’s α reflects the reliability of the variables. The Cronbach’s α value of each construct with multiple items is higher than 0.7, which suggests high internal consistency reliability.

**Table 1:** Confirmatory factor analysis

Variables	Cronbach’s α	Factor loading	AVE	CR
doctor-patient relationship	0.81	0.80-0.91	0.73	0.89
Conflict avoidance	0.85	0.71-0.88	0.63	0.90
Role overload	0.84	0.73-0.86	0.62	0.89
Work engagement	0.88	0.69-0.84	0.58	0.92

The convergent validity was measured by Composite Reliability (CR) and Average Variance Extracted (AVE). As shown in Table 1, the value of CR of each construct is higher than 0.7, and the value of AVE of every construct is greater than 0.5, implying high convergent validity of the measurements.

The indicator factor loading of every item is higher than 0.6, showing that discriminant validity is adequate. In addition, the discriminant validity was also tested by the comparison between the correlations among constructs and square root of the AVE. In Table 2, the values of the square root of AVE are on the diagonal. Each construct’s correlations with other constructs is lower than the square root of AVE scores of this construct, which means a high discriminant validity of the measurements in this study.

**Table 2:** Correlations and discriminant validity

	Variables	Means	S.D.	1	2	3	4	5	6	7	8	9
1	Gender	1.43	0.49	-								
2	Age	2.36	0.65	0.05	-							
3	Marriage	1.58	0.47	0.52^***^	-0.01	-						
4	Education	2.37	0.54	-0.03	0.19^*^	-0.02	-					
5	Doctor qualification	1.06	0.56	0.26^**^	-0.01	0.39^***^	0.02	-				
6	doctor-patient relationship	3.46	0.66	0.14	-0.06	0.16^*^	0.05	0.12	(0.85)			
7	Conflict avoidance	3.56	0.71	-0.02	-0.01	-0.07	-0.17^*^	-0.08	0.07	(0.80)		
8	Role overload	3.48	0.72	0.06	-0.12	0.07	-0.02	-0.03	0.24^**^	0.20^**^	(0.79)	
9	Work engagement	3.71	0.57	0.10	0.01	0.10	-0.06	0.14	0.32^***^	0.23^**^	0.21^**^	(0.76)

Note: *n* =172. *p ≤ 0.05; **p ≤ 0.01; ***p ≤0.001;

### Common method bias

First, we adopted Harman’s one-factor test [56]. The result showed that all items were categorized into four factors, with eigenvalues greater than 1.0, which accounts for 62.39% of the total variance, and the first factor accounts for 27.56% of the variance. Then, we performed confirmatory factor analysis and compared the model fit among the measurement model, one-factor model, and measurement model with method factor [57]. The results showed that the fit of our measurement model (χ^2^[183] = 335.79, CFI =0.91, RMSEA = 0.07, RMR=0.05) was significantly better than the fit of the model with only one method factor (χ^2^[189] = 1097.64, CFI = 0.43, RMSEA = 0.17, RMR=0.12). Also, we included a common method factor in the measurement model. The results of the measurement model with both constructs and a method factor (χ^2^[162] = 286.25, CFI = 0.92, RMSEA = 0.07, RMR=0.04) marginally improved the model fit of the measurement model with only constructs (∆CFI= 0.01, ∆RMR=0.01). The path coefficients and their significance were similar between the two measurement models. Therefore, common method bias is not a serious problem for our study [58].

### Hypotheses tests

To test our hypotheses, we employed hierarchical regression analyses via SPSS 22. We reported the standardized coefficients in Table 3. To reduce the issue of multicollinearity, we mean-centered and standardized the independent and moderator variables before calculating the interaction term [59]. And the variance inflation factors (VIFs) are all below 2.0 in these models, also suggesting that multicollinearity issue is not significant in this study.

**Table 3:** Results of the regression analysis

	Work engagement			Role overload			
Independent variables	Model1	Model2	Model3	Model4	Model5	Model6	Model7
**Controls**							
Gender	0.03	0.01	0.00	0.05	0.03	0.03	-0.00
Age	0.03	0.05	0.07	-0.13	-0.11	-0.11	-0.12
Marriage	0.05	0.01	0.01	0.05	0.02	0.03	0.06
Education	-0.09	-0.10	-0.10	0.01	0.00	0.04	0.03
Doctor qualification	0.14	0.12	0.13	-0.06	-0.08	-0.07	-0.09
Doctor-patient relationship (DPR)		0.31^***^	0.27^**^		0.25^**^	0.23^**^	0.24^**^
Conflict avoidance (CA)						0.19^*^	0.18^*^
Role overload			0.16^*^				
DPR*CA							-0.16^*^
*R*^2^	0.04	0.13	0.15	0.03	0.09	0.12	0.15
∆R^2^	0.04	0.09	0.02	0.03	0.06	0.03	0.03
F	1.10	3.28^**^	3.49^**^	0.77	2.09^*^	2.63^*^	2.86^**^
∆F	1.10	15.79^***^	4.45^*^	0.77	9.77^**^	5.93^*^	4.29^*^

Note: *n*=172. *p ≤ 0.05; **p ≤ 0.01; ***p ≤0.001

#### The relationship between doctor-patient relationship and work engagement

First, we reported all control variables with the dependent variable. Model 1 includes only the control variables (i.e. gender, age, marriage, education, doctor qualification) and explains a relatively small part of the variance in the dependent variable (*R*^2^=0.04). The results of Model 1 showed that gender (β=0.03, *p*>0.05), age (β=0.03, *p*>0.05), marriage (β=0.05, *p*>0.05), education (β=-0.09, *p*>0.05), doctor qualification (β=0.14, *p*>0.05) do not have significant effect on work engagement. Subsequently, we added the independent variable to test the main effect. As predicted, the results of Model 2 in Table 3 shows that doctor-patient relationship is positively related to doctors’ work engagement (β=0.31, *p*≤0.001). Thus, H1 was supported, which means that the better doctor-patient relationship, the higher level of work engagement.

#### The mediating effect of role overload

H2 predicted the effect of doctor-patient relationship on work engagement is mediated by role overload. We used the steps of Baron and Kenny [60]. First, it has been confirmed that doctor-patient relationship is positively related to doctors’ work engagement (β=0.31, *p*≤0.001, Model 2), indicating that the independent variable is significantly related to the dependent variable. In Model 4, the results showed that the control variables do not have significant effect on role overload (gender: β=0.05, *p*>0.05; age: β=-0.13, *p*>0.05; marriage: β=0.05, *p*>0.05; education: β=0.01, *p*>0.05; doctor qualification: β=-0.06, *p*>0.05). Then, doctor-patient relationship is positively related to their role overload (β=0.25, *p*≤0.01, Model 5), suggesting that the independent variable is significantly related to the mediator. Next, we entered role overload as the mediating factor in Model 3, and found that role overload is positively related to work engagement (β=0.16, *p*≤0.05, Model 3), showing that the mediator is significantly related to the dependent variable. Finally, when role overload was incorporated into the regression in which work engagement was the dependent variable, the effect of doctor-patient relationship on work engagement remained significant, but the coefficient of doctor-patient relationship became smaller (β=0.27, *p*≤0.01, Model 3) than coefficient in Model 2 when role overload was not included (β=0.31, *p*≤0.001, Model 2). The results show that the role overload partly mediated the effect of doctor-patient relationship on work engagement. Therefore, H2 was supported.

In addition, to further confirm the mediating effect, we also conducted a bias-corrected bootstrapping procedure [61]. The results were shown in Table 4. The results of bootstrapping suggest that the indirect effect of doctor-patient relationship on work engagement via role overload was significant and positive (95 per cent CI = 0.0013 to 0.0834; excluding 0; indirect effect = 0.0352). Therefore, Hypothesis 2 was supported.

**Table 4:** Indirect effects of doctor-patient relationship on work engagement

Path: Doctor-patient relationship - Role overload - Work engagement	
Bootstrap—indirect effect	0.0352
Standard error	0.0211
Lower limit 95% CI	0.0013
Upper limit 95% CI	0.0834

Note: *n*=172; 5000 resamples

#### The moderating effect of conflict avoidance

H3 proposed that conflict avoidance moderated the relationship between doctor-patient relationship and role overload. We adopted hierarchical moderated regression analyses to test this hypothesis. First we entered control variables and independent variable (i.e. doctor-patient relationship) into the regression. Then the moderator (i.e. conflict avoidance) was incorporated. Finally, we added the interaction between doctor-patient relationship and conflict avoidance into the regression. Results in Table 3 showed that the interaction between doctor-patient relationship and conflict avoidance was negatively related to role overload (β=-0.16, *p*≤0.05, Model 7). Accordingly, H3 was supported.

**Fig. 2:**
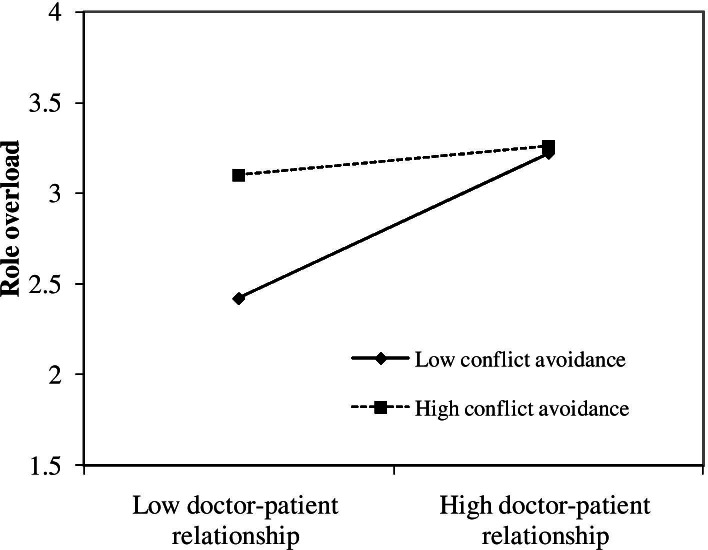
Moderating role of conflict avoidance

To further explore the patterns of the significant interaction effects that supported the hypotheses, we plotted the significant interaction effects using one standard deviation above and below the mean to represent high and low levels of the moderating variables [59]. Figure 2 shows that the slopes are much steeper when conflict avoidance is low than high. We also tested the statistical significance of these two slopes [62]. When conflict avoidance is one standard deviation below the mean, the coefficient of doctor-patient relationship on role overload (β=0.34, t=3.67) is significantly higher than coefficient when conflict avoidance is one standard deviation above the mean (β=0.15, t=1.73). The results confirmed that conflict avoidance negatively moderated the effect of doctor-patient relationship on role overload.

## Discussion and Conclusion

### Discussion

Past studies have shown that doctor-patient relationship can influence health outcomes [63, 64]; however, few researchers have explored the impact and underlying mechanisms of conflicts with regard to residents’ occupational behaviors. Our study’s empirical results showed that maintaining good doctor-patient relationship significantly improved residents’ work engagement. The findings suggest that maintenance of good doctor-patient relationship could trigger a sense of reciprocity among residents and patients, thus leading residents to work better in an effort to meet their patients’ needs. Thus, hospitals can use effective control of doctor-patient conflicts as an important method of achieving improvements and positive results in residents’ professional training. Residents themselves must improve their doctor-patient relationship and reduce conflicts by improving their own clinical ability, communication and interpersonal skills, and so on; for their part, hospitals should build a positive atmosphere for doctor-patient relationship, as this is vital to improve residency programs and training performance [65].

Unexpectedly, we identified role overload as a potential positive mediator between doctor-patient relationship and residents’ work engagement; as the saying goes, “with great power comes great responsibility,” and residents faced with overload may find themselves motivated to engage more deeply and improve their relationships. Role overload variables have been used not only in research dealing with job burnout but also in research related to doctor-patient relationship and work engagement. Although the role overload phenomenon may seem very obvious from the viewpoint of residents, we still need to determine whether these results can be generalized to medical workers beyond the research participants here. Nevertheless, our current results can spur more fundamental theorizing on how role overload can help increase residents’ enthusiasm and energy at work and help residents grow professionally and personally. This approach is more practically meaningful than simply discussing reasons for residents’ job burnout. The conservation of resources theory holds that positive power is the vital motive that allows residents to continue developing in their career lives [66]. This perspective leads to some key questions: how can immature physicians gain a sense of recognition and support from work by dealing with complex doctor-patient relationship; how can they use missions in the work sphere to shape their professional ability; and how can they accumulate experience from interactions with patients in order to build better competence.

Unlike previous research [67, 68], this study treated conflict avoidance as more than just an emotional management strategy for individuals in the medical field, verifying its negative moderating effect on doctor-patient conflicts in the Chinese medical environment. This research used a key forecasting factor and discussed conflict avoidance by proposing and testing a theoretical model under which conflict avoidance affected the relationship between doctor-patient conflict and role overload. The findings expand the scope of research on mechanisms affecting doctor-patient relationship and help explain the function of conflict avoidance with regard to the boundary condition of this relationship in the Chinese cultural background. These researchers’ results show that administration departments are responsible for medical residents should place more attention on individual-level differences in conflict avoidance [9]. For residents with low conflict avoidance, this negative work experience may be even more pronounced. Therefore, we must realize that, at this early stage of standardized residency training in China, we cannot ignore these differences.

### Conclusion

This paper utilized an empirical research method to explore the potential mechanisms underlying the effect of doctor-patient relationship on medical residents’ work engagement and verified the mediating effect of role overload and the moderating effect of conflict avoidance in the model. Doctor-patient relationship in China is facing challenges, and residents are being subjected to overload and overtimes; researchers, educators, and clinical leaders must recognize the complex situations and take effective actions to build good doctor-patient relationship and maintain physicians’ work engagement at an optimum level.

### Theoretical contributions

The theoretical contributions of our study to the literature placed at the intersection between doctor-patient relationship and medical human resource management. Under the conservation of resources theory, we went beyond the well-explored traditional doctor-patient relationship or employee engagement study [28, 29]. This is the first to empirically investigate residents’ employee engagement as an outcome of doctor-patient relationship. Also, very little is known concerning the roles of mediator and moderator variables in the relationship between doctor-patient relationship and residents’ engagement. The empirical model suggests that the effect of doctor-patient relationship on resident behavior is not as straightforward as expected. It cannot be overlooked that what really matters is the role of role overload and conflict avoidance plays. Thus, the results also spur more fundamental theorizing on how doctor-patient interaction serves as a mechanism to integrate residents into clinical practice and to help them to grow.

### Practice implications

The problem of doctor-patient conflicts has been resolved in some successful health organizations; however, until recently, there has been little academic research on the mechanisms underlying non-conflicting doctor-patient relationship. Since the problem-directed research model still forms a core aspect of academic research, it is necessary to develop a clear understanding of why we need to emphasize doctor-patient relationship management and how it can improve residents’ work engagement. We identified factors that could explain residents’ work engagement improvement and stated how these factors can be utilized in Chinese medical settings in the future. The model we created does not include all potential mediating and modifying variables, but it provides a systematic, logical explanation of doctor-patient relationship, physician burnout, work engagement, and mechanisms among them in this setting. This knowledge could be useful in increasing medical departments’ motivation to improve doctor-patient relationship and reduce conflicts; furthermore, it can yield solutions for training departments to improve their training results and, at the same time, provide young physicians with new methods to improve their work engagement and keep good relationships with patients and patients’ families.

The findings suggested that teachers should understand and help each resident build a targeted relationship; guide residents to their best practices for doctor-patient intimacy, appreciation and attention, feedback and criticism, and crisis emotional support. We also suggest that health care policy makers work with residents to develop a common strategy in order to make residents get the most effective interaction for patients, individuals, teams and the residency programs, let residents propose their thoughts on the aspects of assignment arrangements and role behaviors. Integrate doctor-patient relationship building and engagement training into current residency programs, develop specific, measurable and comprehensive performance criteria, in order to make the new requirement of capability operate, and develop a path that helps residents achieve their career ideals and high level of dedication status.

### Limitations

Although this study possesses a number of strengths, there are some limitations. First, the study is cross-sectional in design, thus any causal conclusions drawn should be viewed with caution. Future research could employ a longitudinal study and multi-level data from multiple respondents to extend our findings. Second, the generalizability of this study may be limited because the sample was restricted to Chinese tertiary hospitals from the south-east region and the central region. Hence, it could be recommended that future research should be expanded the sample source and size to address this drawback. Third, although this study considered several objective control variables, future research may include a more comprehensive list of control variables, including training levels, departments, length of time, training satisfaction, and so on. Finally, the findings suggest that good doctor-patient relationship can prompt residents to increase their engagement. To test more potential mechanisms, future research should include self- and other-report of doctor-patient relationship and residents’ work engagement, as well as potential mediators such as big five personality, person-organization fit of residents.

## Data Availability

The datasets used and/or analysed during the current study are available from the corresponding author on reasonable request.

## Abbreviations

ICU: Intensive Care Unit
CFA: Confirmatory Factor Analysis
CR: Composite Reliability
AVE: Average Variance Extracted
VIFs: Variance Inflation Factors
DPR: Doctor-Patient Relationship
CA: Conflict Avoidance
